# A Novel Air-Door Opening and Closing Identification Algorithm Using a Single Wind-Velocity Sensor

**DOI:** 10.3390/s22186837

**Published:** 2022-09-09

**Authors:** Wentian Shang, Lijun Deng, Jian Liu

**Affiliations:** 1 College of Safety Science and Engineering, Liaoning Technical University, Huludao 125105, China; 2 Key Laboratory of Mine Thermo-Motive Disaster & Prevention, Ministry of Education, Huludao 125105, China

**Keywords:** air-door opening and closing, machine learning, tunnel ventilation, wind-velocity data fluctuation, wind-velocity monitoring

## Abstract

The air-door is an important device for adjusting the air flow in a mine. It opens and closes within a short time owing to transportation and other factors. Although the switching sensor alone can identify the air-door opening and closing, it cannot relate it to abnormal fluctuations in the wind speed. Large fluctuations in the wind-velocity sensor data during this time can lead to false alarms. To overcome this problem, we propose a method for identifying air-door opening and closing using a single wind-velocity sensor. A multi-scale sliding window (MSSW) is employed to divide the samples. Then, the data global features and fluctuation features are extracted using statistics and the discrete wavelet transform (DWT). In addition, a machine learning model is adopted to classify each sample. Further, the identification results are selected by merging the classification results using the non-maximum suppression method. Finally, considering the safety accidents caused by the air-door opening and closing in an actual production mine, a large number of experiments were carried out to verify the effect of the algorithm using a simulated tunnel model. The results show that the proposed algorithm exhibits superior performance when the gradient boosting decision tree (GBDT) is selected for classification. In the data set composed of air-door opening and closing experimental data, the accuracy, precision, and recall rates of the air-door opening and closing identification are 91.89%, 93.07%, and 91.07%, respectively. In the data set composed of air-door opening and closing and other mine production activity experimental data, the accuracy, precision, and recall rates of the air-door opening and closing identification are 89.61%, 90.31%, and 88.39%, respectively.

## 1. Introduction

The air-door is a device that is used for adjusting the volume and direction of air flow in a mine ventilation system [1]. Air flow turbulence during the air-door opening and closing process can cause abnormal fluctuations in the wind-velocity sensor data in the associated roadway, resulting in false alarms from the wind-velocity sensors [2,3]. Thus, the opening and closing of an air-door in a mine is as important as a door opening and closing in a building for fire prevention and control [4,5,6]. Therefore, timely identification of the opening and closing of the air-door is critical.

**Figure 1:**
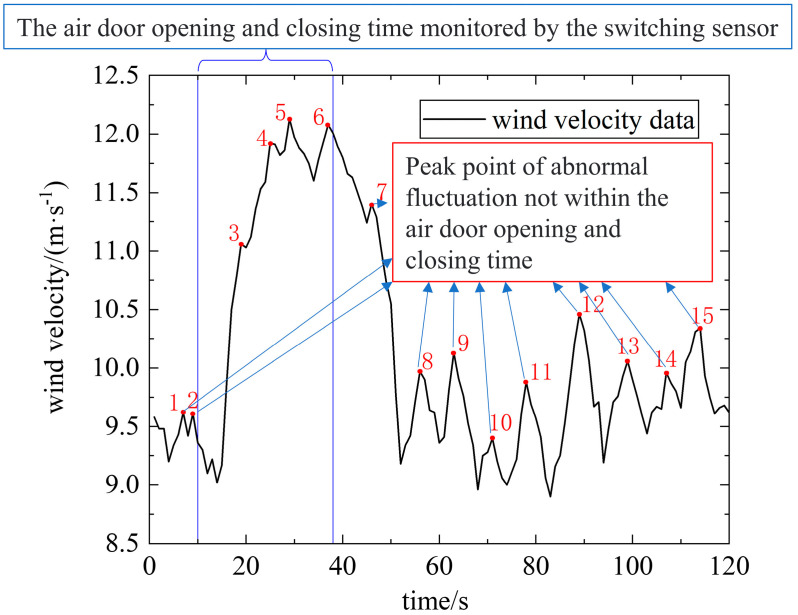
Wind-velocity sensor data containing the air-door opening and closing process.

In the absence of interference from production activities, the wind-velocity sensor data are not smooth lines; they show irregular small amplitude fluctuations under the action of turbulent pulsation. Under the joint action of the air-door opening and closing and turbulent pulsation, the abnormal fluctuation time of the wind-velocity data will appear outside the air-door opening and closing time. Our research group proved this fact through similar experiments, as shown in Figure 1.

As can be seen from Figure 1, there is no obvious correlation between the multiple abnormal fluctuations in the wind-velocity sensor data and the air-door opening and closing time monitored by the switching sensor. Therefore, the air-door opening and closing status monitored by the switching sensor can only be used to determine whether the abnormal fluctuation of the wind-velocity data during the opening and closing time is caused by the air-door (and not whether the abnormal fluctuation of the wind-velocity data outside the opening and closing time is caused by the air-door). If these abnormal fluctuations are determined by field personnel, abnormal fluctuations in the wind-velocity caused by other production activities or safety accidents before or after the air-door opening and closing may be ignored. The wind-velocity sensor data for identifying the opening and closing state of the air-door can be used to clarify whether the abnormal fluctuation is caused by the opening and closing of the air-door, so as to avoid other production activities and safety accidents caused by ignoring the abnormal fluctuation of the wind speed.

Because the number of wind-velocity sensors in the mine is less than the number of tunnels, and the wind-velocity sensor data in the mine is affected by turbulence pulsation, and the data fluctuation in the air-door opening and closing process is strongly influenced by the occurrence time, duration, and opening and closing angles of the air-door, such identification is difficult using regular statistics. 

In the field of mine ventilation, no special algorithm is available for identifying the opening and closing of the air-door. Existing algorithms mainly focus on fault identification, such as roadway collapse, wall collapse, and air-door damage [7,8,9]. They can only identify long-term changes in the wind resistance or volume from large amounts of sensor data. Owing to the short air-door opening and closing time, and the small number of wind-velocity sensors that generate data changes during the process, the fault identification algorithms may fail. To address this issue, the identification of air-door opening and closing must be based on a new theory and an algorithm framework must be constructed in accordance with its characteristics. Wind-velocity sensor data constitute a sequence of wind-velocity data indexed in time order, i.e., a type of time-series data. Hence, time-series data anomaly identification methods from other engineering fields can be referenced. For rotating machinery, blast furnace iron-making, power quality signals, pipeline transportation, and other fields, Yao et al. proposed a gear fault diagnosis method based on the time and frequency-domain signals and a convolutional neural network (CNN) model [10]. Vununu et al. proposed a drill-bit fault diagnosis method based on power spectrum density (PSD) images and a deep convolutional autoencoder (DCAE) [11]. Glowacz designed a fault detection method for electric impact drills and coffee grinders using the root mean square (RMS), MSAF-17-MULTIEXPANDED-FILTER-14, and nearest neighbor (NN) classifier [12]. Wang et al. proposed a method for identifying unknown faults in the iron-making process using variable selection and a moving-window hidden Markov model (VS-MWHMM) [13]. Zhou et al. proposed a fault identification method for molten iron quality using monitoring indicator functions and kernel partial least squares (KPLS) [14]. Ouyang et al. introduced a fault detection and identification method for the blast furnace iron-making process using a gated recurrent unit (GRU) network and support vector data description (SVDD) [15]. Kikuta proposed an interference detection method for high-precision ranging using frequency dependence characteristics and adaptive notch filters [16]. Yang et al. proposed a method for detecting power quality waveform abnormalities using discrete wavelet transform (DWT) and residual singular values (RSV) [17]. Wang et al. proposed a method for classifying power quality disturbances using a CNN [18]. Mishra reviewed power quality disturbance detection and classification methods based on digital signal processing (DSP) and machine learning [19]. Jia et al. proposed a method for classifying abnormal pipeline working conditions using hoop strain information and support vector machines (SVM) [20]. Jia et al. developed a method for locating leakage points along a pipeline using wavelet packet vectors and support vector regression (SVR) [21]. Priyanka et al. studied the identification of failure rates in oil transportation pipelines using an unsupervised machine learning technique and a partition clustering algorithm [22]. Xu et al. proposed a method for identifying pipeline leaks using a dynamic threshold identification method (DTIM) and a Raman distributed fiber sensor (RDFS) system [23].

From the aforementioned studies, we find that the main idea of time-series data anomaly identification methods is identification using machine learning after deep mining of the data features. There are many feature mining methods and machine learning models for time-series data anomaly identification. Popular feature mining methods include basic statistics (BS) [24], fast Fourier transform (FFT) [25], short-time Fourier transform (STFT) [26], continuous wavelet transform (CWT) [27], DWT [28], and auto-regressive moving average (ARMA) [29]. Popular machine learning models include SVM [30], k-nearest neighbor (KNN) [31,32], gradient boosting decision tree (GBDT) [33,34], Bayesian network (BN) [35], decision tree (DT) [36], random forest (RF) [37], and CNN [38]. 

Accordingly, an algorithm for air-door opening and closing identification based on a single sensor is proposed. As DWT is a mature feature extraction method, it overcomes the shortcoming of FFT or STFT whereby the time-frequency resolution cannot be met at the same time. Compared with CWT or the semi-orthogonal wavelet transform (SWT), DWT has less redundancy [39], and the global information and fluctuation information of the data can be obtained through coordination with statistical features. Therefore, DWT and statistical features were chosen as the feature extraction methods for this algorithm. As the machine learning model in the algorithm, this study employs a similar experimental method for evaluating the performance of the algorithm under the SVM, GBDT, BN, and RF models. To better evaluate the effectiveness of the model, the accuracy rate (AC), precision rate (PR), and recall rate (RE) of the air-door opening and closing time identification are used as indicators to analyze the differences between the models, so as to determine the machine learning model that makes the best use of the mined data features and obtains the best opening and closing time results.

## 2. Identification Algorithm

This section describes the construction of the air-door opening and closing identification method using a single wind sensor. It consists of four parts. First, the total workflow of the proposed identification method is introduced. Second, the preprocessing step, including discrete normalization and multi-scale sliding window discretization, is described in detail. Third, the training of the classification model is described comprehensively. Finally, the merging and selection step is introduced. 

To enhance the readability of the article, we include a nomenclature list as shown in Table 1. 

### 2.1. Workflow of the Method

**Figure 2:**
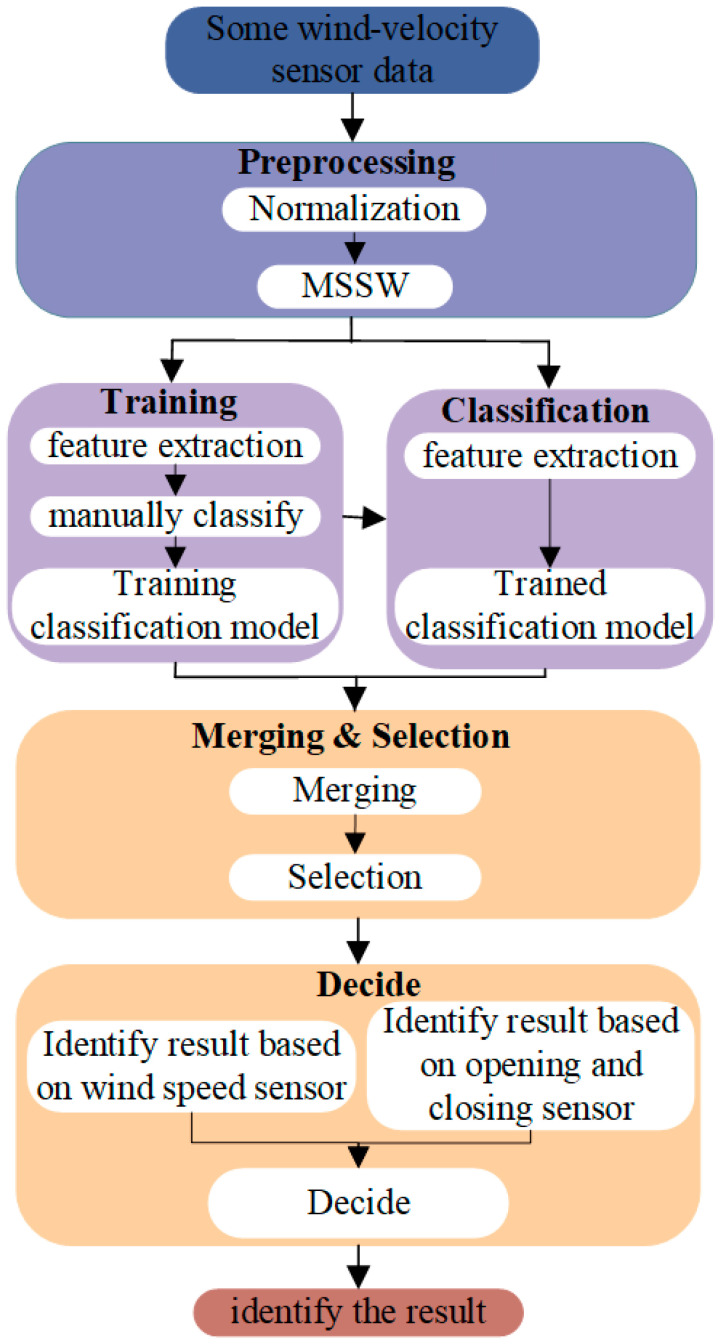
Flowchart of the air-door opening and closing identification method.

Figure 2 shows the flowchart of the proposed method. The proposed method comprises four main steps.

**Step 1:** Preprocessing. The variation range of various pieces of wind-velocity sensor data is uniformly reduced to [0,1] via normalization. Then, a multi-scale sliding window is used to generate an original sample (G) consisting of sub-time-series data. Finally, the original sample is divided into the training sample ($G^{r}$) and the test sample ($G^{e}$) in the ratio of 7:3.

**Step 2:** Training. The feature vectors of each sub-time-series data in the training and test samples are extracted using traditional statistics and DWT. Then, the sub-time-series data categories of the training samples are classified, and the categories and feature vectors corresponding to the sub-time-series data of the training samples are used for training the classification model. 

**Step 3:** Classification. The sub-time-series data feature vectors of the test sample are used as the input of the trained classification model, and the model classifies them into categories.

**Step 4:** Merging and Selection. The sub-time-series data of each scale belonging to the air-door opening and closing set ($G_{r}^{e}$) are merged using the union method. After the merging is completed, the optimal air-door opening and closing time-series data are selected.

### 2.2. Preprocessing

Normalization and discretization are well-known techniques in data preprocessing [40]. Therefore, this section describes the normalization and discretization methods and the specific data change process of the algorithm in detail.

#### 2.2.1. Normalization

Data normalization can be used to convert wind-velocity data from different ranges into data in the range [0,1], which is convenient for the subsequent unified processing. In this study, the wind-velocity sensor data are processed using deviation standardization, which is expressed as follows:

(1) $$x\prime =\frac{\left(x-x_{\min}\right)}{\left(x_{\max}-x_{\min}\right)}$$

where x′ represents the normalized data; x represents the wind-velocity sensor data; $x_{\min}$ represents the minimum value of the wind-velocity sensor data; and $x_{\max}$ represents the maximum value of the wind-velocity sensor data.

The *k*-th piece of the wind-velocity monitoring data $\{X_{k}|x_{i}^{k}\},i=1,2,\ldots ,L$, after deviation standardization, changes to $\{X\prime_{k}|x\prime_{i}^{k}\},i=1,2,\ldots ,L$.

#### 2.2.2. Discretization

To identify the air-door opening and closing time with different range more effectively, we propose a discrete processing method for one-dimensional wind-velocity sensor data based on the multi-scale sliding window method commonly used in image identification [41]. Based on the Coal Mine Safety Regulations in China and the data characteristics of the wind-velocity sensor data, the parameter constraints are obtained. The parameters selected for the multi-scale sliding window are governed by the following four constraints:

(2) $$Q_{min}\leq S\leq Q_{max}$$

(3) $$Q_{max}\cdot Z\leq w$$

(4) $$P_{min}\leq w\leq P_{max}$$

(5) $$\left\{\begin{array}{l} t_{j}=\frac{w}{2}\;l_{j}>=\frac{w}{2}\\ t_{j}=l_{j}\;l_{j}<\frac{w}{2} \end{array}\right.$$

Equation (2) represents the constraint on the number of scaling times, where S is the number of scaling times, and $Q_{min}$ and $Q_{max}$ represent the minimum and maximum number of scaling times, respectively. $Q_{min}$ is greater than or equal to 2. Equation (3) represents the constraint on the scaling ratio and scaling times, where Z is the scaling ratio and w is the set of the sliding window scale. Equation (4) represents the constraint on the sliding window scale, where $P_{min}$ and $P_{max}$ represent the minimum and maximum scale of a reasonable sliding window, respectively. $P_{min}$ is greater than or equal to 2, and $P_{max}$ is less than the shortest air-door opening and closing time. Equation (5) represents the constraint on the sliding distance, where $t_{j}$ is the sliding distance of the w-scale sliding window, and $l_{{}^{j}}$ is the remaining length of the time-series data after the w-scale sliding window has been slid j times.

According to the four aforementioned constraints, we select the number of scaling times as 4, the scaling ratio as 2, and the sliding window size as 4. When these parameters are used, the *k*-th piece of the normalized wind-velocity sensor data $\{X\prime_{k}|x\prime_{i}^{k}\},i=1,2,\ldots ,L$ is discretized through a multi-scale sliding window to generate multiple sub-time-series data, and each time-series data is expressed as follows:

(6) $$\left\{\begin{array}{l} T_{k,j}^{s}=\{t_{2j-1}^{s},t^{s}{}_{2j},t^{s}{}_{2j+1},t^{s}{}_{2j+2}\}\;2j+2\leq L^{s}\\ T_{k,j}^{s}=\{t_{L^{s}-3}^{s},t^{s}{}_{L^{s}-2},t^{s}{}_{L^{s}-1},t^{s}{}_{L^{s}}\}\;2j+2>L^{s} \end{array}\right.$$

where $T_{k,j}^{s}$ represents the sub-time-series data in the j-th sliding window after division s of the *k*-th piece of wind-velocity sensor data. Further, $t_{2j-1}^{s},t^{s}{}_{2j},t^{s}{}_{2j+1},t^{s}{}_{2j+2}$ denote the series data contained in $T_{k,j}^{s}$.

**Figure 3:**
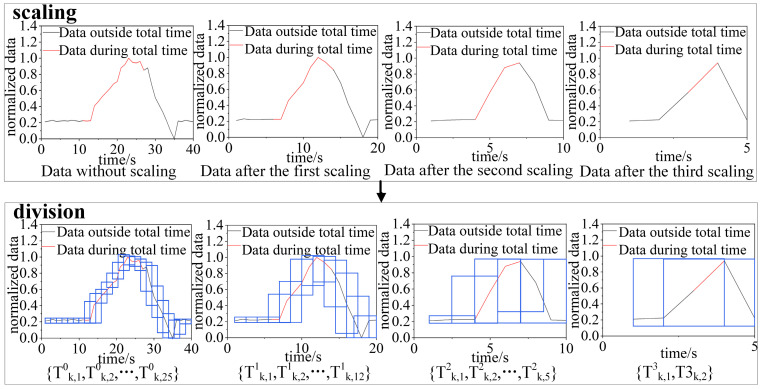
Wind-velocity sensor data discretization process.

The discretization process for a piece of wind-velocity sensor data is shown in Figure 3.

### 2.3. Training

After all the wind-velocity sensor data is preprocessed, the original samples G composed of the sub-time-series data are generated, and the test samples $G^{e}$ and training samples $G^{r}$ are divided in a ratio of 7:3. It should be noted that the sub-time-series data belonging to the same segment of the sensor data must belong to the same data set. This section describes how the feature vectors are extracted from each sub-time series using traditional statistics and DWT, as well as the classification rule.

#### 2.3.1. Extracting Features Based on Traditional Statistics

In terms of traditional statistical feature extraction, this study selects four statistical features as global features of the sub-time-series feature vectors, as shown in Table 2.

#### 2.3.2. Extracting Features Based on DWT

DWT involves the representation of a signal with a finite length or a fast-decaying oscillatory waveform that is scaled and panned to match the input data, with good time-frequency local analysis capability and multi-resolution analysis characteristics. The process of DWT of sub-time-series data can be expressed as follows:

(7) $$WT_{f}(q,w)=\int {}_{R}f(t)\cdot \bar{\psi_{q,w}(t)}\;dt$$

where f(t) denotes a sub-time-series data; $WT_{f}(q,w)$ is the sub-time-series data as a result of DWT; t is the sequence of data; q is a scale parameter; w is the translation parameter along the time axis; and $\psi_{q,w}(t)$ is a wavelet base function. In this study, the db1 wavelet is used.

After processing via DWT, the sub-time-series data are decomposed into several layers, with each layer consisting of high-frequency and low-frequency coefficients. The fluctuation features are obtained by performing entropy sum calculations on these high-frequency or low-frequency coefficients. The entropy sum formula for any layer of low-frequency and high-frequency coefficients is calculated as follows:

(8) $$\left\{\begin{array}{l} e_{js}=\sum_{i=1}^{n_{s}}-cs_{ji}\times \log_{2}^{cs_{ji}}\\ e_{jd}=\sum_{i=1}^{n_{d}}-cd_{ji}\times \log_{2}^{cd_{ji}} \end{array}\right.$$

where $e_{js}$ represents the entropy sum of the low-frequency coefficients in layer j; $e_{jd}$ represents the entropy sum of the high-frequency coefficients in layer j; $cs_{ji}$ represents the i-th low-frequency coefficient in layer j; $cd_{ji}$ represents the i-th high-frequency coefficient in layer j; $n_{s}$ represents the total number of low-frequency coefficients in layer j; and $n_{d}$ represents the total number of high-frequency coefficients in layer j.

Therefore, this study uses a db1 wavelet with a filter length of 2; hence, the number of decomposition layers is 2 and the number of fluctuating features obtained is 4.

#### 2.3.3. Feature Vector and Classification

According to Section 2.3.1 and Section 2.3.2, each sub-time-series feature vector consists of statistical features and fluctuating features. The feature vector $C_{k,j}^{s}$ of the sub-time-series data $T_{k,j}^{s}$ is expressed as follows:

(9) $$C_{i}^{k}={\left(\bar{x\prime },x\prime_{min},x\prime_{max},\sigma^{2},e_{1s},e_{1d},e_{2s},e_{2d}\right)}^{T}$$

Based on the known air-door opening and closing time for each piece of wind-velocity sensor data, all the time-series data are divided into two categories, namely, the sequence data within the air-door opening and closing time and the sequence data outside the air-door opening and closing time, which are used as the training sets of the classification model after classification.

### 2.4. Merging and Selection

After classifying the sub-time-series data in the test set using the trained model, we can obtain the optimal complete time-series data by merging and selecting the sub-time-series data. This section describes the merging process of the sub-time-series data of different scales belonging to the category of the air-door opening and closing time and the selection process of the merging results of each scale in detail.

#### 2.4.1. Sub-Time-Series Merge

**Figure 4:**
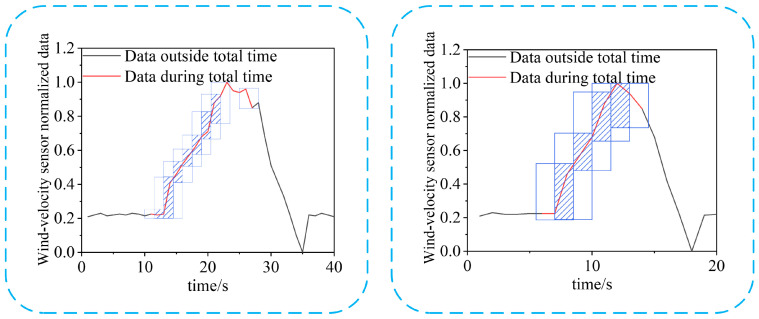
Example of sub-time-series data of different samples before merging.

The sub-time-series data belonging to the air-door opening and closing time category on each scale of the wind-velocity sensor data are part of the complete air-door opening and closing time-series data. There may be some overlap among the parts, as shown in Figure 4. In the figure, the data in each blue box is the sub-time-series data belonging to the category of the air-door opening and closing time, whereas the shaded part is the overlapping part of two sub-time-series data. Therefore, we need to judge the overlap and merge the two sub-time-series.

Intersection over Union (*IoU*) is used to judge the overlap between two sub-time-series data. The *IoU* between two sub-time-series data can be calculated as follows:

(10) $$IoU_{ab}=\frac{T_{k,a}^{s}\cap T_{k,b}^{s}}{T_{k,a}^{s}\cup T_{k,b}^{s}}$$

where $T_{k,a}^{s}$ and $T_{k,b}^{s}$ represent two sub-time-series data.

When *IoU* > 0, the two time-series data overlap. The union method is used to merge the two sub-time-series data. The merging of the two overlapping sub-time-series data and the confidence calculation of the merged sub-time-series data can be expressed as follows:

(11) $$\left\{\begin{array}{l} T_{k,m}^{s}=T_{k,a}^{s}\cap T_{k,b}^{s}\\ c_{k,m}^{s}=\frac{c_{k,a}^{s}+c_{k,b}^{s}}{2} \end{array}\right.$$

where $T_{k,m}^{s}$ represents the merged sub-time-series data; $c_{k,m}^{s}$ represents the confidence of the merged sub-time-series data; and $c_{k,a}^{s}$ and $c_{k,b}^{s}$ represent the confidence of $T_{k,a}^{s}$ and $T_{k,b}^{s}$, respectively.

Based on the overlapping judgment and the merging of the two time-series data, a method for merging all the sub-time-series data belonging to the air-door opening and closing time of each piece of the wind-velocity sensor data is proposed. The merging process of all the sub-time-series data belonging to the air-door opening and closing time category of a piece of the wind-velocity sensor data is shown in Algorithm 1.
**Algorithm 1.** Merging process of time-series data of time identification samples.**Inputs: Four scales of sub-time-series data belonging to the air-door opening and closing time category****Outputs: Merged time-series data belonging to the air-door opening and closing time category**1. for s in 0, 1, 2, 3 do2.    f, d, y = 03.    i = 14.    for x in [x_i_, …, x_i+1_] do5.     calculate $IoU_{x,x+1}$ by Equation (11)6.     if $IoU_{x,x+1}>0$ then7.       if f = d then8.         merge $T_{k,x}^{s}$$\mathrm{and}\;T_{k,x+1}^{s}$→$T_{k,y}^{s}\prime$ by Equation (10)9.         d = d + 110.        end if11.        if f < d then12.          merge $T_{k,x}^{s}$$\;\mathrm{and}\;T_{k,x}^{s}$→$T_{k,y}^{s}\prime$ by Equation (10)13.        end if14.     end if15.     if $IoU_{x,x+1}\leq 0$ then16.        {}$\;\mathrm{append}\;T_{k,y}^{s}\prime$17.        f = f + 118.        y = y + 119.    end if20.   end for21.    i = i + 222. end for

#### 2.4.2. Optimal Time-Series Data Selection Based on *IoU* and Confidence

**Figure 5:**
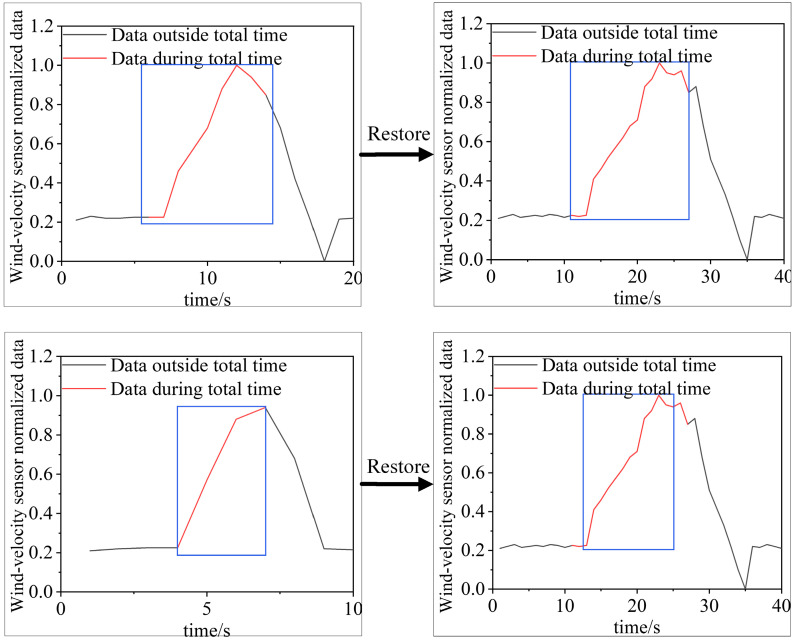
Examples of time-series data restoration at different scales.

For a section of the wind-velocity sensor data, the time-series data of each scale belong to the category of the air-door opening and closing time $\left\{T_{k,0}^{0}\prime ,\ldots ,T_{k,y_{1}}^{0}\prime ,T_{k,y_{2}}^{1}\prime ,\ldots ,T_{k,y_{3}}^{1}\prime ,T_{k,y_{4}}^{2}\prime ,\ldots ,T_{k,y_{5}}^{2}\prime ,T_{k,y_{6}}^{3}\prime ,\ldots ,T_{k,y_{7}}^{3}\prime \right\}$. The scaled time-series data are restored as shown in Figure 5.

The recovered sub-time-series data are the preliminary identification results. Because there is more than one preliminary result, an optimal time-series selection method based on *IoU* and confidence is proposed.

In this method, the *IoU* threshold is used to eliminate the incorrect results. First, we set an *IOU* threshold. If the average *IOU* value of a result is smaller than the threshold, the result is eliminated. In this paper, the *IOU* threshold is 0.1. After eliminating the incorrect result, the result with the highest confidence is selected as the optimal identification result. The average *IoU* of each identification result and the other identification results is calculated as follows:

(12) $$\bar{IoU_{k,m}}=\frac{\sum_{n=1}^{n_{m}}\frac{T_{k,m}^{s}\cap T_{k,n}^{s}}{T_{k,m}^{s}\cup T_{k,n}^{s}}}{n_{m}}$$

where $\bar{IoU_{k,m}}$ represents the average *IoU* of $T_{k,m}^{s}$ time-series data and other time-series data, and $n_{m}$ represents the number of time-series data in the preliminary result.

**Figure 6:**
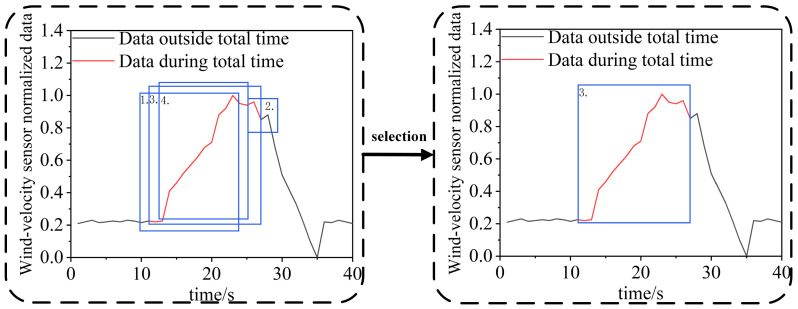
Example of preliminary identification results.

After the optimal selection, only one initial identification result of a section of the wind-velocity sensor data is retained, as shown in Figure 6.

## 3. Similar Experiments

This section describes a similar experimental model designed to carry out some experiments. It consists of two parts. First, we describe the design principle of the similarity model and the construction of the experimental platform. Second, we describe the experimental conditions and summarize some experimental data laws.

### 3.1. Design and Construction of the Experimental Platform

Owing to the complex conditions in the mine, field tests of air-door opening and closing may lead to disturbances in the flow field around the air-door and affect the normal production of the mine. When there is a working face in the roadway associated with the air-door, it may cause a gas explosion or other accidents that would endanger the safety of mine workers. In addition, the abnormal fluctuation of wind speed data caused by the air-door opening and closing may lead to abnormal fluctuation of the velocity sensor only in the associated tunnel. Therefore, this study designs a similar experimental platform to collect velocity data.

**Figure 7:**
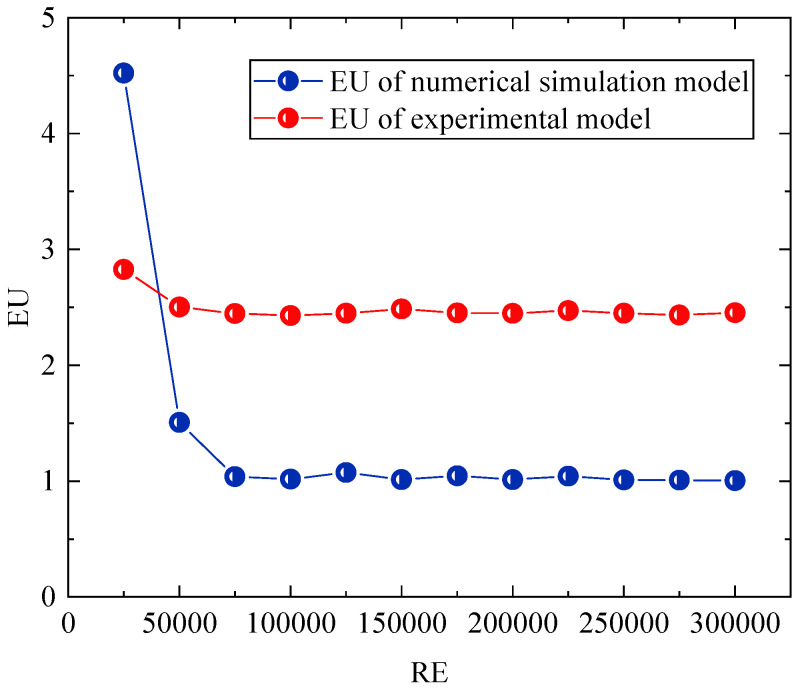
Curves of EU variation with RE within the numerical simulation model and the experimental model.

The experimental model of the prototype design refers to the experimental tunnel of the Key Laboratory of Mine Thermodynamic Disasters and Control of the Ministry of Education of Liaoning University of Engineering and Technology. According to the literature [42,43,44], within two geometrically similar models, the flow field enters a second self-simulation zone when the Euler number (EU) is independent of the Reynolds number (RE), satisfying the flow similarity principle. The overall similarity ratio between the experimental model and the experimental roadway is 1:16, and the length direction variation is 2, so as to meet the geometric similarity [42]. To meet the flow similarity, a numerical simulation is conducted to obtain the EU variation curves under different RE, as shown in Figure 7.

**Figure 8:**
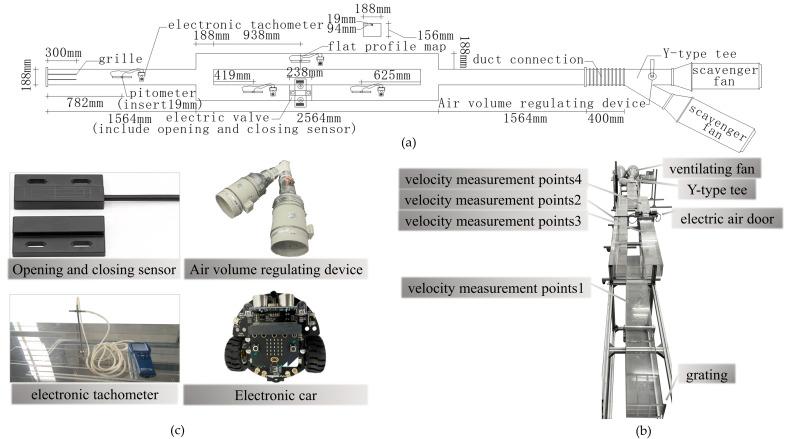
Experimental system: (**a**) experimental size and principle; (**b**) sensor used in the experiment and other experimental equipment for other mine production activities; (**c**) experimental system entity diagram.

As shown in Figure 7, the EU of both the numerical model and the numerical simulation model basically do not change when the RE is greater than 0.75 × 10^5^. When the inlet wind-velocity of the experimental model is greater than 7.9 m/s, the flow is similar to that of the experimental roadway when the inlet wind-velocity is greater than 0.49 m/s. After determining the parameters of the experimental model as described above, the experimental system of the damper opening and closing is obtained, which is composed of the roadway model, an electric damper, a velocimeter, and a fan, as shown in Figure 8. Figure 8a shows the size and principle of the experimental system, Figure 8b shows the sensor used in the experiment and other experimental equipment for other mine production activities, and Figure 8c shows the entity diagram of the experimental system.

In the experimental device shown in Figure 7, the switching sensor is used to determine the air-door opening and closing time in each experiment, and the wind-velocity adjusting device of the electronic car and fan are used to carry out an experiment on the airflow disorder caused by the running of the mine car and the wind-velocity adjustment.

### 3.2. Experimental Conditions and Laws

#### 3.2.1. Air-door Opening and Closing Experiment

The four variable parameters of the air-door opening and closing experiment are as follows: parameter 1, inlet wind-velocity; parameter 2, air-door opening and closing velocity; parameter 3, air-door opening and closing angle; and parameter 4, air-door fixed angle opening time. Under the premise that the air-door starts opening at 10 s, experiments were conducted for 336 working conditions by arranging and combining the different values of the four parameters listed in Table 3.

**Figure 9:**
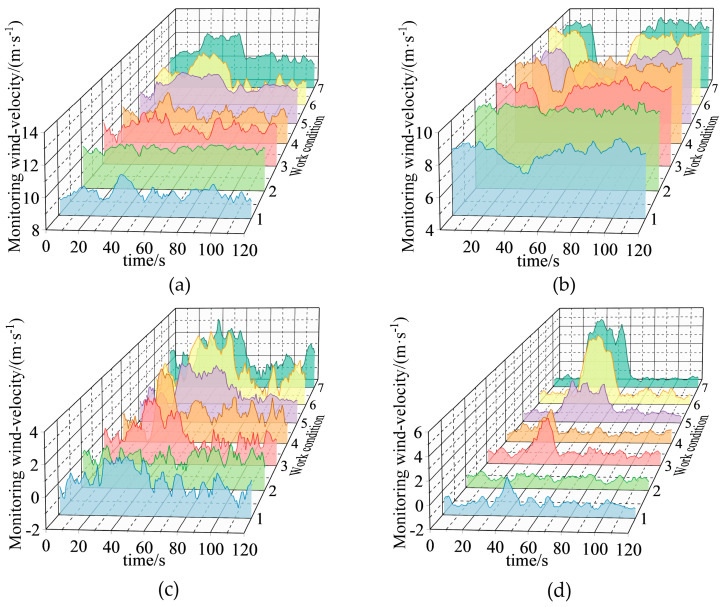
Variation of wind-velocity data in a part of the air-door opening and closing experiments: (**a**) monitoring point 1; (**b**) monitoring point 2; (**c**) monitoring point 3; and (**d**) monitoring point 4.

Some working conditions are summarized in Table 4, and their data are shown in Figure 9.

As shown in Figure 9, when there is no air-door disturbance, the wind-velocity data of each measuring point also fluctuate, owing to the existence of turbulent pulsation. The wind-velocity data at different monitoring positions fluctuate more significantly during the opening period of the air-door, and the measuring points at the inlet roadway, the ventilation side of the air-door, and the windward side of the air-door show an upward trend. The wind-velocity data of parallel roadway measurement points in the roadway where the air-door is located shows a downward fluctuation trend.

#### 3.2.2. Other Mine Production Activity Experiment

Other mine production activity experiments include two types of production activities: one is the mine car running experiment, and the other is the fan wind-velocity adjusting experiment.

The three variable parameters of the mine car running experiment are as follows: parameter 1, inlet wind-velocity; parameter 2, mine car running velocity; parameter 3, mine car running direction. Under the premise of the same size, running distance, and number of wind-velocity sensors, the different values of the three parameters listed in Table 5 are permuted and combined, and 36 types of working conditions are considered. The experiment is repeated three times for each working condition.

The three variable parameters of the fan wind-velocity adjusting experiment are as follows: parameter 1, inlet wind-velocity; parameter 2, adjust the wind-velocity; parameter 3, adjust the time of the wind-velocity. Under the premise of the same time of wind-velocity adjustment, different values of the three parameters listed in Table 6 are permuted and combined to obtain a total of 60 working conditions, and the experiment is repeated three times for each working condition.

**Figure 10:**
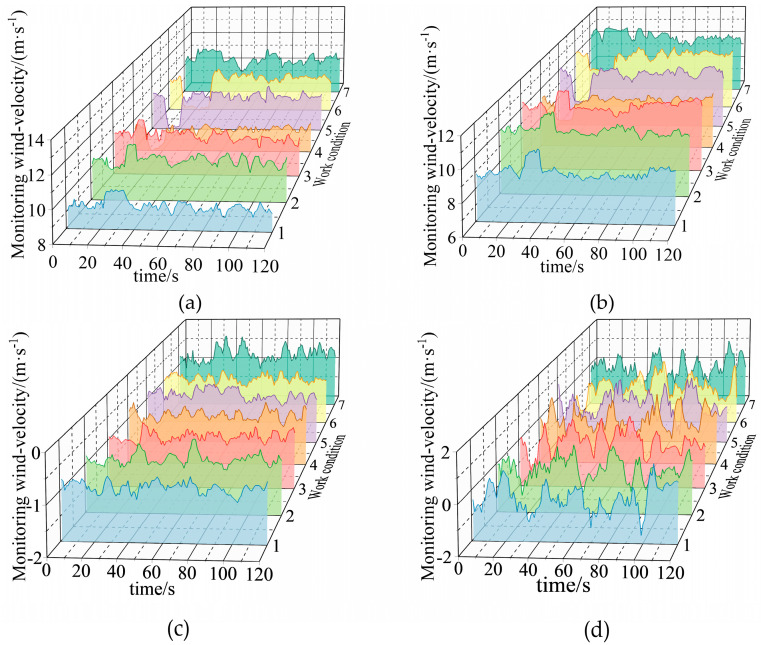
Variation of wind-velocity data in a part of the other mine production activity experiments: (**a**) monitoring point 1; (**b**) monitoring point 2; (**c**) monitoring point 3; and (**d**) monitoring point 4.

Some working conditions are summarized in Table 7, and their data are shown in Figure 10.

As shown in Figure 10, mine car running and fan wind-velocity adjustment will also lead to obvious fluctuations in the wind-velocity data of monitoring point 1 and monitoring point 2.

## 4. Validation and Comparison

### 4.1. Machine Learning Protocol

Scikit-learn (sklearn) is a powerful machine learning library provided by a third party (Python). Based on the open-source code of sklearn, we constructed a novel air-door opening and closing identification algorithm using a single wind sensor by employing SVM, GBDT, BN, and RF.

### 4.2. Evaluation Index

To verify the identification effect of the proposed method on the air-door opening and closing time, we choose three indicators, namely accuracy (AC), precision (PR), and recall (RE). The average value of these indicators in each air-door opening and closing stage is used to evaluate the effect of the opening and closing stage division. They can be calculated using the true positive (TP), true negative (TN), false negative (FN), and false positive (FP). They are expressed as follows:

(13) $$AC=\frac{TP+TN}{TP+FP+TN+FN}$$

(14) $$PR=\frac{TP}{FP+TP}$$

(15) $$RE=\frac{TP}{TP+FN}$$

### 4.3. Model Selection and Algorithm Effect

In this section, the effectiveness of the algorithm is verified using the data set composed of the experimental data of the air-door opening and closing. Because there are other production activities besides the air-door opening and closing in the actual mine, this study used the data set composed of the experimental data of the air-door opening and closing and the experimental data of other mine production activities to validate the algorithm.

#### 4.3.1. Experimental Data of Air-door Opening and Closing Are Used to Verify the Effect of the Algorithm

This study used 1344 experimental data including 336 air-door opening and closing conditions and 4 velocity measurement points were used as the dataset; 70% of the data set was used as the training set for the classification model, and the remaining 30% was used as the test set for the overall method. To avoid bias, 10 folds cross-validation were performed.

**Figure 11:**
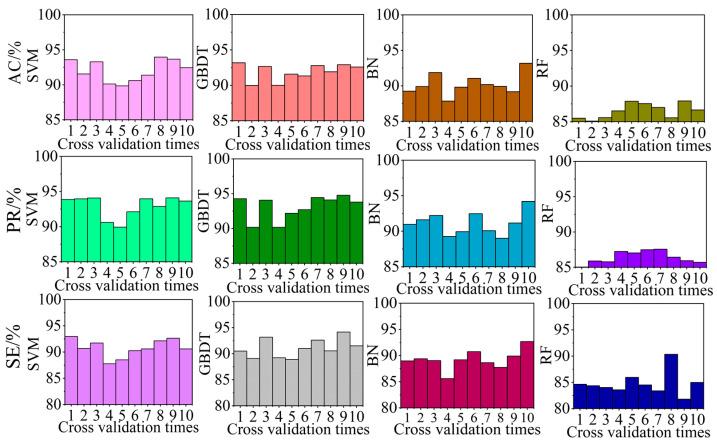
Identification effect of different models used in the algorithm.

To select the optimal classification model, we compared the identification effects of the air-door opening and closing identification algorithm based on four different classification models, namely SVM, GBDT, BN, and RF. The test set identification effect of each cross-validation using the identification algorithm with different classification models is shown in Figure 11. The average identification effect of the test set for 10 folds cross-validation using the identification algorithm with different classification models is summarized in Table 8.

According to Figure 11 and Table 8, when the GBDT model is employed, the AC, PR, and RE of the algorithm are more than 90%, which meet the requirements of practical engineering applications. The recognition effect is better than that of the algorithm using the other models. The AC, PR, and RE of the algorithm using the RF model are all less than 90%, and the identification effect in not as good as that of the algorithm using the other models. When the SVM model is employed, the AC, PR, and RE of the algorithm are more than 90%; however, the identification effect is not as good as that of the GBDT model. When the BN model is employed, only the PR exceeds 90%. Therefore, the classification models employed in the air-door opening and closing recognition algorithm can be ranked on the basis of the recognition effect as follows: GBDT > SVM > BN > RF.

#### 4.3.2. Experimental Data of Air-door Opening and Closing Used to Verify the Effect of the Algorithm

**Figure 12:**
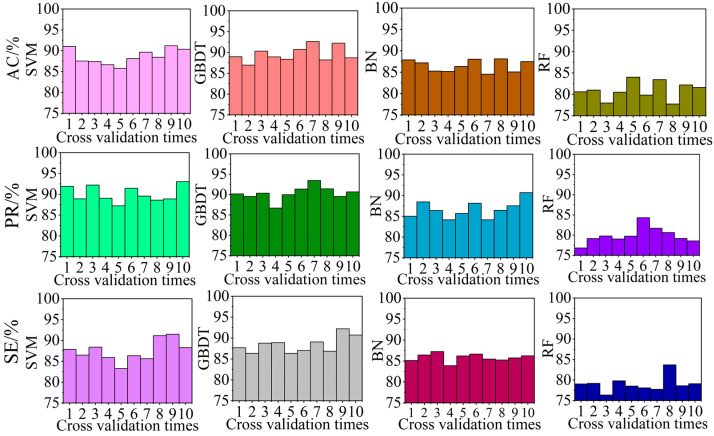
Identification effect of different models used in the algorithm.

In this section, the wind speed data of 360 car running experiments are added to the data set used in Section 4.3.1, and the proportion of the air-door opening and closing experimental data in each cross-validation process is the same as that of other mine production activities. The test set identification effect of each cross-validation using the identification algorithm with different classification models is shown in Figure 12. The average identification effect of the test set for 10 folds cross-validation using the identification algorithm with different classification models is summarized in Table 9.

According to Figure 12 and Table 9, when there are other production activity experiments in the data set, the recognition effect of the algorithm will decrease. At this time, the PR, and RE of the algorithm using the RF model are all less than 80%, and the recognition effect is not as good as that of the algorithm using other models. When the BN model is used, the AC, PR, and RE of the algorithm are all less than 87%, and the recognition effect is only better than that of the algorithm using RF. When SVM is used, although the PR of the algorithm reaches 90%, the AC and RE of the algorithm are less than 90%. When GBDT is used, the PR of the algorithm both reach 90%, and the AC and RE of the algorithm also reaches 88%. Therefore, when there are other production activity experiment data in the data set, the identification effect of the GBDT model is still the best.

## 5. Conclusions

(1) It is difficult to identify the abnormal fluctuation of wind speed caused by the air-door opening and closing in the complex ventilation system of a mine, and the wind-velocity sensors may raise false alarms. To overcome this problem, we proposed an air-door opening and closing recognition algorithm. The algorithm innovatively uses the monitoring data of a single wind-velocity sensor as the original data, extracts the data features via statistical indicators and DWT, and then classifies the data using a machine learning algorithm. Finally, according to the classification results, efficient identification of the air-door opening and closing time is realized.
(2) The effectiveness of the proposed algorithm was verified via similar experiments. Through comparison and verification, when GBDT was selected as the machine learning model in the algorithm, the algorithm exhibited the best recognition effect for the air-door opening and closing time. When the test data set is composed entirely of the air-door opening and closing experiment data, the AC, PR, and RE of the algorithm are 91.81%, 92.99%, and 90.53%, respectively. When the test data set is composed entirely of the air-door opening and closing experiment data and other mine production activity experiment data, the AC, PR, and RE of the algorithm are 89.61%, 90.31%, and 88.39%, respectively.
(3) If the algorithm is applied in mines with subsequent improvements, it can effectively identify whether the abnormal wind speed fluctuation is caused by the opening and closing of the air-door through the wind speed data of a single sensor, and can distinguish the abnormal wind speed data fluctuation caused by other production activities and the opening and closing of the air-door. It can effectively solve the false alarm problem of wind speed sensor and reduce the workload of field staff.
(4) The proposed algorithm is suitable for the identification of not only air-door opening and closing but also other wind-velocity disturbance activities in mines. In the future, we will try to modify the algorithm so that it can be used for the running position identification of the mining car and other problems that remain to be solved.
(5) This study has the following limitation. Only four common machine learning algorithms were analyzed, namely SVM, GBDT, BN, and RF. Deep learning methods such as CNN will be further studied in the future.
(6) As is well known, there is no universal best method. In this study, only machine learning algorithms were used to identify the opening and closing of the air-door in a mine. The conclusions drawn from this study may not be applicable when addressing other issues. It is necessary to comprehensively analyze the nature of the problem to be solved as well as the principle of various machine learning algorithms, and subsequently select the most appropriate algorithm in order to obtain the optimal solution.
(7) We plan to conduct a large-scale field test in the mine after improving the algorithm, so as to verify its potential for applications effect of the algorithm in an actual mine.

## Figures and Tables

**Table 1:** Nomenclature list.

Names and Abbreviations	Meaning
Air-door opening and closing	This process consists of three stages: the air-door from closing to opening to an angle, the air-door stays open at this angle for a period of time, and the air- door stays open at this angle to fully closed.
MSSW (multiple-scale sliding window)	A computer vision technique that can detect objects at multiple scales
Sub-time series data	Sliding-window partition of time series data
DWT (discrete wavelet transformation)	A method for time and scale analysis of data
SVM (support vector machines)	A machine learning model that classifies data by finding a plane that separates the two sides
Integrated learning	The idea of combining multiple weak learners to form a strong one
Boosting	A school of integrated learning in which integrated weak learners are interdependent
Bagging	Another school of integrated learning, integrated weak learners do not depend on each other
GBDT (gradient boosting decision tree)	An addition model based on boosting ensemble learning
BN (Bayesian network)	A model extended from the Bayes method
RF (random forest)	A model based on bagging ensemble learning idea
IoU (Intersection over Union)	A test criterion derived by dividing the overlapping part of two regions by the set part of the two regions
AC (accuracy rate)	The ratio of the correct quantity recognized by the algorithm to the total correct quantity
PR (precision rate)	The proportion of the target category samples identified by the algorithm that are really the target category samples
RE (recall rate)	The ratio of the target category samples identified by the algorithm to all the target category samples

**Table 2:** Four statistical features.

Statistical Features	Expression
mean value	$\bar{x\prime }=\sum_{i}^{k}x\prime_{i}/k$
minimum value	$x\prime_{min}=\min(S_{i}^{k})$
maximum value	$x\prime_{max}=\max(S_{i}^{k})$
variance	$\sigma^{2}=\sum_{i}^{k}\left(x\prime_{i}-\bar{x\prime }\right)/k$

**Table 3:** Specific parameters of each component of the air-door opening and closing factors.

Parameter	Unit	Values
1	m/s	8.5, 9.5, 10.5
2	°/s	3, 5, 10, 15
3	°	45, 60, 75, 90
4	s	10, 15, 20, 25, 30, 35 40

**Table 4:** Setting of each parameter condition in a part of the air-door opening and closing experiments.

Working Condition	Parameter 1/(m/s)	Parameter 2/(°/s)	Parameter 3/(s)	Parameter 4/(°)
1	9.5	5	10	45
2	10.5	0	0	0
3	10.5	5	10	45
4	10.5	15	10	45
5	10.5	5	30	45
6	10.5	5	10	90
7	10.5	15	30	90

**Table 5:** Specific parameters of each component of the mine car running factors.

Parameter	Unit	Values
1	m/s	8.5, 9.5, 10.5
2	m/s	0.04, 0.06, 0.08, 0.10, 0.12, 0.16
3	None	With wind, against wind

**Table 6:** Specific parameters of each component of the fan wind-velocity adjusting factors.

Parameter	Unit	Values
1	m/s	8.5, 9.5, 10.5
2	m/s	8, 9, 10, 11
3	s	5, 10, 15, 20, 30

**Table 7:** Setting of each parameter condition in a part of the other mine production activity experiments.

Working Condition	Production Activity	Parameter 1	Parameter 2	Parameter 3
1	mine car running	9.5 m/s	0.04 m/s	with wind
2	mine car running	10.5 m/s	0.08 m/s	with wind
3	mine car running	10.5 m/s	0.12 m/s	against wind
4	fan wind-velocity adjusting	9.5 m/s	8 m/s	10 s
5	fan wind-velocity adjusting	10.5 m/s	8 m/s	10 s
6	fan wind-velocity adjusting	10.5 m/s	9 m/s	20 s
7	fan wind-velocity adjusting	10.5 m/s	11 m/s	20 s

**Table 8:** Average identification effect of all folds cross-validation when different models are used in the algorithm.

Index	SVM	GBDT	BN	RF
AC/%	92.04	91.89	90.22	86.52
PR/%	92.91	93.07	91.09	86.40
RE/%	90.79	91.07	89.19	84.76

**Table 9:** Average identification effect of all folds cross-validation when different models are used in the algorithm.

Index	SVM	GBDT	BN	RF
AC/%	88.60	89.61	86.50	80.89
PR/%	90.09	90.31	86.68	79.89
RE/%	87.48	88.39	85.82	79.02

## Data Availability

Not applicable.
